# Surface oxidation of petroleum pitch to improve mesopore ratio and specific surface area of activated carbon

**DOI:** 10.1038/s41598-020-80784-2

**Published:** 2021-01-14

**Authors:** Song Mi Lee, Seon Ho Lee, Doo-Hwan Jung

**Affiliations:** 1 Fuel Cell Laboratory, Korea Institute of Energy Research (KIER), Daejeon, 34129 Republic of Korea; 2 Advanced Energy and System Engineering, University of Science and Technology (UST), Daejeon, 34113 Republic of Korea; 3 Department of Chemical and Biomolecular Engineering, Yonsei University, Seoul, 03722 Republic of Korea

**Keywords:** Materials science, Nanoscience and technology

## Abstract

In this study, surface oxidation of petroleum pitch was performed to enhance the thermal stability, specific surface area, and mesopore ratio of activated carbon. The oxygen uptake of the pitch by surface oxidation has a strong influence on the formation of the specific surface area and pore size of activated carbon. It was confirmed that the oxygen uptake from the surface to the inner side of the surface oxidized pitch was the highest at the temperature of 330 °C (IP330-AC), with a mesopore ratio of 63.35% and specific surface area of 1811 m^2^ g^−1^. The oxygen content of the surface oxidized pitch increased proportionately with the mesopore ratio in activated carbon. The specific surface area and mesopore ratio of IP330-AC were respectively 163% and 487% higher than those of petroleum-based commercial activated carbon (A-BAC), and 102% and 491% higher than those of coconut-based commercial activated carbon (P60).

## Introduction

Activated carbon is a functional carbon material having a high specific surface area owing to a large amount of nanospace^1–5^. It is fabricated by a physical activation using steam or chemical activation method, and the shape and surface characteristics of the nanospace can be controlled^6^. The International Union of Pure and Applied Chemistry defines pores with a diameter of less than 2 nm as micropores, between 2 and 50 nm as mesopores, and more than 50 nm as macropores. Generally, activated carbon is a surface functional material and exhibits a bulk and granular carbon structure with a high specific surface area of several hundred to several thousand m^2^ per gram owing to the formation of numerous micropores as well as mesopores with diameters of 50 nm or less^7–9^. However, the critical disadvantages of microporous carbon materials include slow material mobility resulting from space constraints due to small pore sizes, poor electrical conductivity due to enormous surface functionalities, and fragility of the pore structure under heat treatment at high temperature^10^. Activated carbon with mesopores has emerged as a solution to these problems.

Coconut shell is generally used as the raw material for activated carbon^11,12^. However, it is relatively expensive. Therefore, new materials are needed to realize inexpensive activated carbon. Wood-based activated carbon has a higher specific surface area and mesopore content than coconut shell-based activated carbon, but its carbon content and durability are low. Coal-based activated carbon has higher carbon content, pore size, and mesopore content than coconut shell-based activated carbon^13^. Petroleum pitch is a common carbonaceous material and is well known as a good precursor of activated carbon. Moreover, it usually costs less than $100 per ton and is widely available owing to its use as a combustion fuel^14^.

However, it is difficult to produce activated carbon from petroleum pitch because it has a low softening point (SP). One method to help the petroleum pitch endure the high activation temperature is to use surface oxidation. Surface oxidation of pitch has been widely studied. While analyzing the pyrolysis mechanism of coal tar pitch, Grzyb et al. found that surface oxidation of the coal tar pitch is an excellent method for improving thermal stability^15^. Zhichang et al. mentioned that surface oxidation need to maintain the shape of the pitch during activation^16^. Fathollahi et al. explained the effect of surface oxidation on crack formation on the surface^17^. Wasalathanthri et al. explained that surface oxidation of the pitch improve both mesopore ratio and specific surface area^18^. In this study, we intend to determine how surface oxidation of petroleum pitch affects the formation of mesopores in activated carbon and increases the specific surface area.

## Materials and methods

### Raw material

Petroleum pitch with SP of 270 °C, 0.3 wt.% of moisture, 0 wt.% of ash, 52 wt.% of volatile matter, and 47.7 wt.% of fixed carbon was obtained from GS Caltex, Korea. It was used to produce mesoporous activated carbon. The elemental analysis was conducted using TruSpec Elemental Analyzer (LECO Co., USA) and the sulfur content was measured with SC-432DR Sulfur Analyzer (LECO Co., USA).

### Preparation of surface oxidized pitch

The petroleum pitch was crushed and sieved to a size of 500–710 μm. The surface oxidized pitch was heated to 270, 300, 330, and 360 °C at the rate of 2 °C min^−1^ under dry airflow of 100 mL min^−1^ for 2 h. The prepared samples were named as IP270, IP300, IP330, and IP360, respectively.

### Preparation of mesoporous activated carbon

Mesoporous activated carbons were prepared from the surface oxidized pitch by steam activation method. About 5 g of the surface oxidized pitch was then activated at 850 °C for 240 min under steam (250 cm^3^ min^−1^) at the rate of 3 °C min^−1^. For uniform steam activation and efficient reaction, a 59.5 × 3.5 cm jar-shaped rotary kiln furnace was used, which was rotated at the speed of 200 rpm. The prepared activated carbons according to the surface oxidation temperature were named as IP270-AC, IP300-AC, IP330-AC, and IP360-AC.

### Characterization

The materials were further characterized by elemental analysis and proximate analysis. Bruker Alpha-T FT-IR spectrophotometer was applied to identify the functional groups in the pitch surface in the wavenumber range of 400–4000 cm^−1^. The mass changes were evaluated and differential scanning calorimetry (DSC) analysis of the samples was conducted along with thermogravimetric analysis (TGA) using a 449 F3 NETZSCH SDT system to confirm the thermal stability and thermal behavior of the surface oxidized pitches. To confirm the progress of surface oxidation, the oxygen uptake from the surface to the inside was confirmed by scanning electron microscopy energy dispersive x-ray spectroscopy (SEM–EDS) line scanning (Scanning Electron Microscope, LEO-1530, Carl Zeiss, Germany). A surface area analyzer (BELSORP-max, BEL JAPAN, Inc.) was used for the surface area and pore size measurements. The total pore volume (*V*_t_) was calculated from the nitrogen adsorbed at a relative pressure of 0.99. The micropore volume and pore size distribution were calculated from the measured isotherms by using the nonlocal density functional theory model for slit-shaped carbon pores. The mesopores were analyzed by using the BJH (Barrett-Joyner-Halenda) method. The mesopore ratio is calculated by ratio of the mesopore (2–50 nm) volume/total pore volume (*V*_m_ /*V*_t_)^19^.

## Results and discussion

### Analysis of surface oxidized pitch

Activation is required to produce activated carbon from pitch. Steam activation requires a temperature of 800 to 900 °C. Generally, pitch melts at above 400 °C and cannot be steam activated^20^. In this study, the pitch had an SP of 270 °C, and it was necessary to increase the thermal stability and to maintain the granule shape during steam activation, which was achieved by oxygen uptake to the surface^16,21^. The temperature of surface oxidation was up to SP + 90 °C based on the SP, and the pitch was surface oxidized. After the surface oxidation, the amount of oxygen uptake was confirmed through elemental analysis. Table 1 shows the characteristics of the surface oxidized pitch at various temperatures. The C, H, N, and S contents were confirmed by elemental analysis and the O content was calculated as O = 100 − C − H − N − S. The O content of the petroleum pitch was 3.6 wt.%, and the total amount of oxygen uptake of various surface oxidized pitch was confirmed based on this. The surface oxidized pitches did not show the presence of N and S regardless of the reaction conditions. The O content of IP330 was 19.2 wt.% and the amount of oxygen uptake was 15.6 wt.%. As the surface oxidation reaction temperature increases, the reactivity become high, which increases the amount of oxygen uptake. At this time, the surface oxidation reaction occurs two reactions simultaneously: one is the infusibilization reaction in which oxygen is introduced, and the other is the combustion reaction in which oxygen reacts with carbon and escapes in the form of CO or CO_2_. Because the surface oxidation caused by the bonding with oxygen and the combustion in oxygen are simultaneously performed, the amount of oxygen and oxygen uptake in the reaction are not proportional^21^. If the pitch is thick, it takes a long time for the oxygen to penetrate to the inside. Moreover, high reaction energy is required. Generally, it is necessary for the surface oxidation to occur at a high temperature in order to provide a sufficient level of reaction energy to produce the surface oxidized pitch.

**Table 1:** Elemental analysis of surface oxidized pitch at various surface oxidation temperatures.

Samples	C (wt.%)	H (wt.%)	N (wt.%)	S (wt.%)	O (wt.%)	O_intr._*
Petroleum pitch	91.5	4.8	0.1	0.0	3.6	
IP270	84.5	3.9	0.0	0.0	11.6	8.0
IP300	82.6	3.5	0.0	0.0	13.8	10.3
IP330	77.7	3.2	0.0	0.0	19.2	15.6
IP360	78.9	3.1	0.0	0.0	18.0	14.4

*Amount of oxygen uptake.

**Figure 1:**
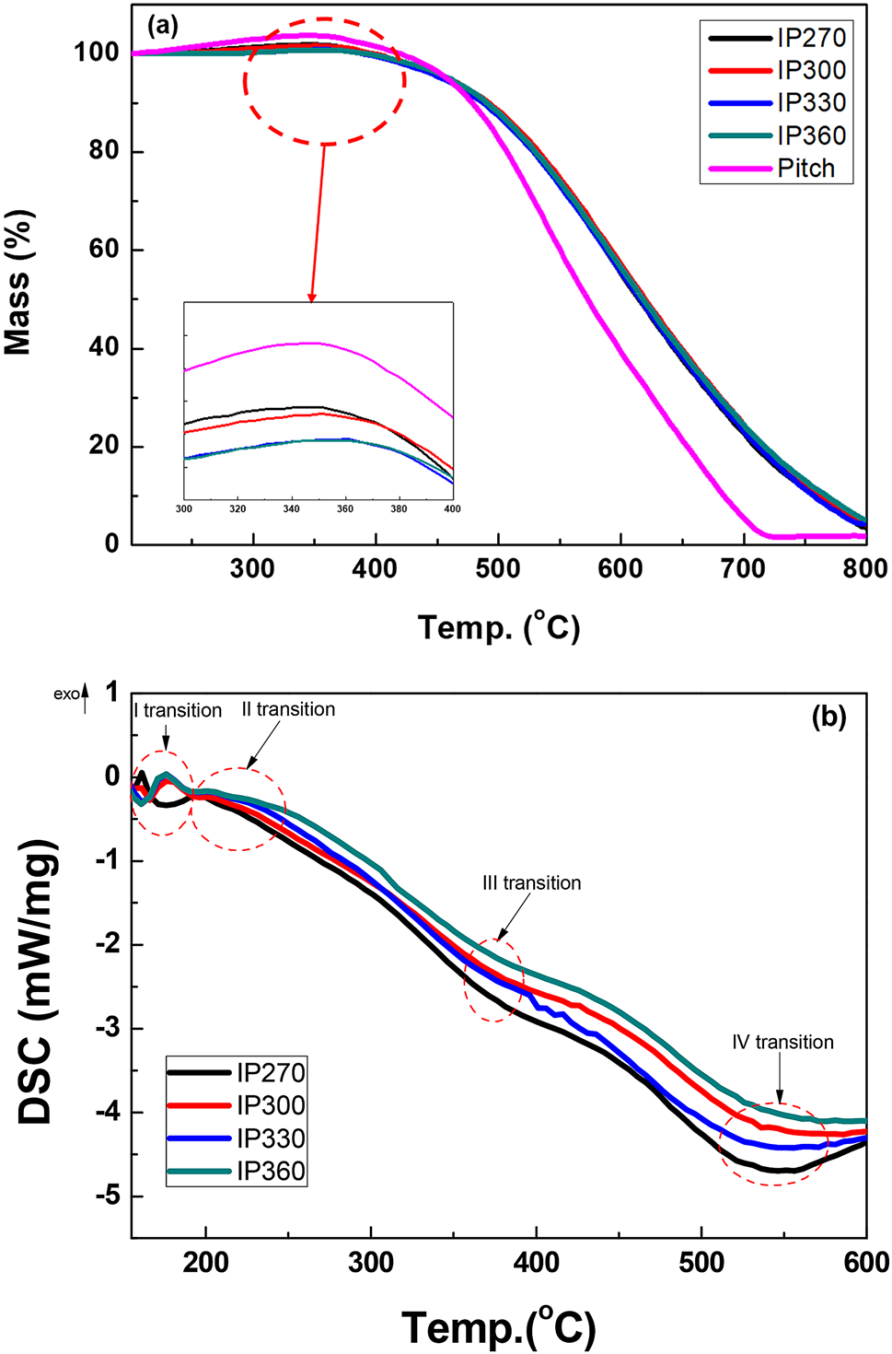
(**a**) TGA, (**b**) DSC results of the surface oxidized pitch at various surface oxidation temperatures.

Surface oxidation of the pitch involves the formation of carbonyl groups and thermal stabilization. This results in the property that its shape does not change even at high temperatures. Figure 1 shows the thermal characteristics of the pitch surface oxidized at various temperatures when heated to 900 °C. The reactions that occur during the surface oxidation process of the pitch are oxidation, dehydration, condensation, crosslinking, and removal^20^. Yoon et al. developed a simple method to monitor and optimize the surface oxidation process through thermal analysis^22^. Experiments at various temperatures showed that the amount of oxygen uptake on the surface of the pitch was the highest at a surface oxidation reaction temperature of 330 °C. On the other hand, the amount of oxygen uptake in IP360 was 14 wt.%, which was lower than that of IP330. IP360 showed a smaller amount of oxygen uptake than IP330, as the combustion reaction was excessive due to the higher reaction energy than the energy need for the infusibilization, resulting the oxygen was burn and emitted to the outside. As a result, as shown in Fig. 1a, the weight of the surface oxidized pitch in the oxygen atmosphere increases to 330 °C, and then decreases afterwards. The TGA curve shows the temperature at which the pyrolysis of each pitch begins. The mass loss of IP270 begins at 360 °C, whereas that of IP360 begins at 370 °C. The residual amount of IP270 at 800 °C was 2.4% higher than that of IP360. The mass losses of the surface oxidized pitch at below 782 °C are related to the volatilization of the pitch component owing to the decomposition of hydrocarbons of various molecular masses. As a result, volatile matter evaporates during heat treatment at various temperatures^23^. Table 2 shows the transition characteristics of each step in the DSC curve. It is confirmed that the surface oxidized pitch has high thermal stability. The endothermic DSC peak between 200 and 600 °C is associated with the decomposition of hydrocarbons of various molecular masses. Different starting temperatures for the decomposition by surface oxidation indicate the presence of hydrocarbon groups of low molecular mass that are more easily volatilized to gaseous form during annealing. Volatile matters are bound to lactone or carbonyl groups through surface oxidation, which increases thermal stability. Four endothermic peaks are observed in the DSC curve in Fig. 1b. The surface oxidized pitch is divided into increasing states of viscosity—melting state, decomposition state, and finally the decomposition end state—as the temperature increases^24^. As shown in Fig. 1, volatile matter is released from the surface oxidized pitch at temperatures between approximately 300 °C and 800 °C. As shown in Table 2 (Transition I), the lowest temperature at which evaporation of volatile matter occurs is 156 °C for IP270, and the remaining samples show a similar pattern. In Transition II, IP270 began to melt at 176 °C and it can be assumed that surface oxidation progressed slowly. Therefore, a surface oxidation temperature of at least 300 °C is required for steam activation of the petroleum pitch.

**Table 2:** Phase change temperature from DSC analysis.

Samples	I. Endothermic transition – from brittle to viscoelastic state			II. Endothermic transition – melting			III. Endothermic transition – decomposition	IV. Endothermic transition – end of decomposition
	Onset °C	End °C	Peak °C	Onset °C	End °C	Peak °C	Onset °C	Onset °C
IP270	156	176	161	176	206	196	326	489
IP300	166	192	176	196	244	204	335	519
IP330	166	191	177	194	247	203	333	513
IP360	162	191	176	193	247	204	338	526

**Figure 2:**
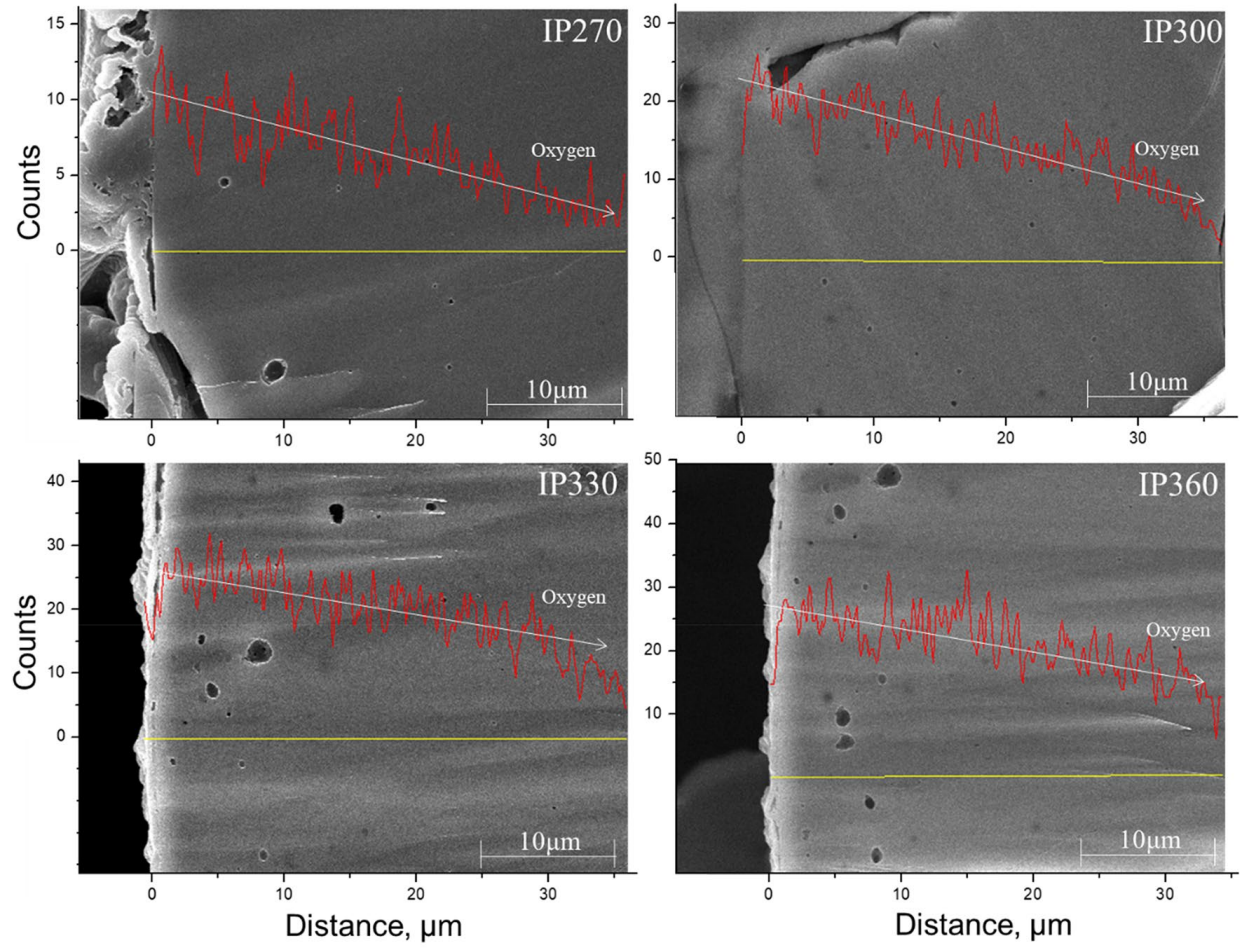
Oxygen uptake of surface oxidized pitch at various surface oxidation temperatures.

Figure 2 shows the oxygen uptake of the surface oxidized pitch at various surface oxidation temperatures with SEM–EDS line scanning analysis^25^. Surface oxidation proceeds from the surface of the pitch to the inside. However, the reaction energy of IP270 was inadequate owing to the low surface oxidation temperature, and oxygen uptake of the pitch surface was not achieved. When the surface oxidation temperature was higher than 300 °C, surface oxidation proceeded better than in IP270, and it was confirmed that oxygen was penetrated adequately to the depth of 30 µm from the surface. On the other hand, the oxygen content on the surface of IP360 decreases and the distribution of oxygen is overall uneven. Table 1 shows that not only the oxygen uptake of the pitch surface but also the gasification reaction in which oxygen is consumed is actively performed.

### Characteristics of surface oxidized pitch-based activated carbon

**Figure 3:**
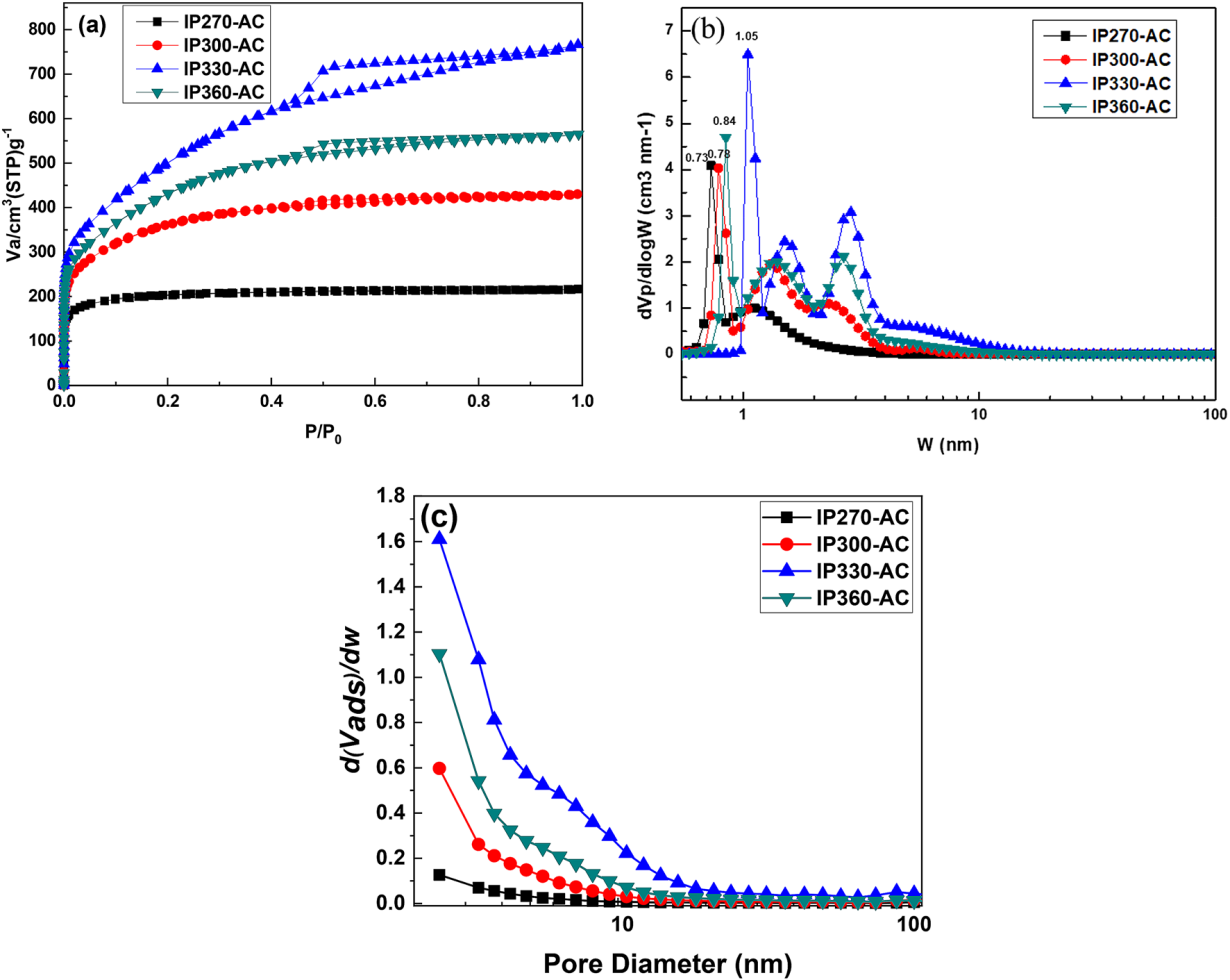
N_2_ adsorption–desorption isotherms at 77 K (**a**) and pore size distribution of petroleum pitch-based activated carbon calculated by (**b**) NLDFT model and (**c**) BJH model.

It is well known that the adsorption capacity of activated carbon can be recognized by its physical characteristics such as porosity, pore-volume, surface area, and pore size^26^. The N_2_ adsorption–desorption isotherm was determined to observe these physical characteristics. Figure 3a shows the N_2_ adsorption–desorption isotherm at 77 K of activated carbon obtained at various surface oxidation temperatures. IP270-AC shows the typical curve of activated carbon with many micropores. The amount of adsorption increased in the order of IP270-AC < IP300-AC < IP360-AC < IP330-AC. In addition, IP330-AC and IP360-AC exhibited the broadest mesoporous structures, where the structures were clearer for IP330-AC than for IP360-AC.

Mochida et al. proved that the surface oxidation of pitch is effective in increasing the specific surface area of activated carbon^27^. As can be seen from IP330, which is an appropriate temperature for surface oxidation, mesopores were formed by the surface oxidation and activation of the pitch, resulting in increased porosity. However, IP360-AC manufactured at high reaction temperature shows that some of the oxygen uptake did not contribute to the increase of specific surface area. As shown in IP360-AC, at a high reaction temperature, the entire oxygen uptake does not contribute to the increase of the specific surface area, but is discharged in the form of CO, CO_2_, and H_2_O, and the walls between the pores are vaporized during the activation process and the pores decrease.

Table 3 compares the physical properties of activated carbon produced by steam activation in the surface oxidized pitch at various temperatures. The specific surface area of the activated carbon prepared according to the surface oxidation temperature varied greatly. Among them, IP330-AC had the highest specific surface area of 1811 m^2^/g and the mesopore ratio was the highest at 63.35%. As shown in Fig. 2, the IP330 was well oxidized by oxygen uptake from the surface to the inside, which affected the high specific surface area and mesopore ratio. Overall, the mesopore ratio of activated carbon prepared with surface oxidized pitches was as high as 13.46–63.35%. IP330-AC had 163% and 102% higher specific surface area, and 487% and 491% higher mesopore ratio, than A-BAC and P60, respectively. It was confirmed that the method is very suitable for the production of mesoporous activated carbon. The isotherm shows sharp adsorption at very low relative pressures (P/P0 < 0.01), which indicates that the microporosity of the sample consists mainly of micropores. However, as oxygen uptake increases, the number of mesopores increases. Figure 3 shows the pore size distribution calculated by (b) NLDFT model and (c) BJH model. As shown in Fig. 3b, c, activated carbon from the pitch with low oxygen content is microporous, but mesopores gradually develop as the oxygen content increases, indicating an increase in the total pore volume and average pore diameter. Specifically, broad peak was appeared at mesopore width range of 5–10 nm after surface oxidation reaction.

**Table 3:** Comparison of physical properties of activated carbon prepared by steam activation of the surface oxidized pitch at various oxidation temperatures.

Samples	*S*_BET_^a^ (m^2^ g^-1^)	*V*_t_^b^ (cm^3^ g^-1^)	*V*_m_^c^ (cm^3^ g^-1^)	Mesopore ratio (%)
IP270-AC	767	0.33	0.05	13.46
IP300-AC	1301	0.67	0.23	34.73
IP330-AC	1811	1.18	0.75	63.35
IP360-AC	1553	0.87	0.43	49.28
* P60	1779	0.93	0.12	12.90
**A-BAC	1114	0.48	0.06	13.02

^a^*S*_BET_: Specific surface area calculated by the Brunauer–Emmett–Teller (BET) method (*P/P*_*0*_ = 0.05–0.15).

^b^*V*_t_: total pore volume.

^c^*V*_m_: mesopore volume.

*P60: Commercial activated carbon (Kuraray, Japan).

**A-BAC: Commercial activated carbon (Kureha, Japan).

Steam activation produces simultaneous gasification and oxidation reaction on the surface. In this case, the reaction formula is expressed by the following equation and both endothermic reactions occur in Eqs. (1) and (2)^28^.

1 $${\text{C}} + {\text{H}}_{{2}} {\text{O}} = {\text{CO}} + {\text{H}}_{{2}} - {29}.{\text{4 kcal}}$$

2 $${\text{C}} + {\text{2H}}_{{2}} {\text{O}} = {\text{CO}}_{{2}} + {\text{2H}}_{{2}} - {19}.0{\text{ kcal}}$$

In addition, the oxygen uptake during surface oxidation also reacts with CO or CO_2_ through the activation process, and pores are formed in the place of the released oxygen, which is advantageous for the formation of mesopores. However, when the surface oxidation temperature is more than 360 ℃, it is difficult to maintain the lactone and carboxyl structure because of an excessive gasification reaction, and it is difficult to effectively form the specific surface area by collapsing before the activation reaction. The specific surface area of IP360-AC is 1553 m^2^ g^−1^, which is smaller than that of IP330-AC. In addition, the surface oxidation at excessively high temperature decreases the amount of oxygen uptake from 15.6% to 14.4% and negatively affects the production of efficient activated carbon. It was confirmed that the oxygen uptake changes depending on the temperature of the surface oxidation and this has a strong influence on the formation of the specific surface area of activated carbon.

**Figure 4:**
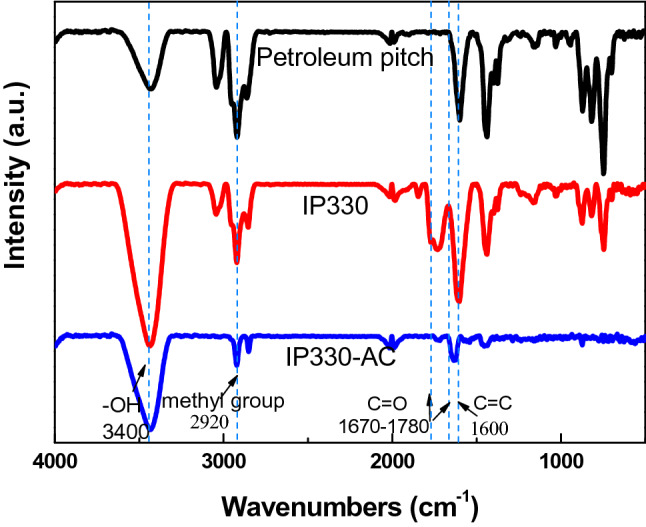
FT-IR spectra of petroleum pitch, surface oxidized pitch, and activated carbon.

Figure 4 shows the FT-IR spectra of the oxygen uptake of surface oxidized pitch and changes in the oxygen functional groups through steam activation. The spectra of all samples show broad hydrogen-bonding peaks of -OH in the region of 3400 cm^−1^. At 2920 cm^−1^, where the aliphatic methyl group appears, the sharp peak of the petroleum pitch becomes smaller after surface oxidation and reduces further after activation. This is due to the removal of aliphatic materials such as methyl groups, which are relatively lighter than the aromatics, and the effect is exacerbated by the gasification reaction occurring during the heat treatment at each stage. Removal of the aliphatic material results in the formation of pores, which increases the specific surface area. In the carbon compound, an atomic group in which C in the middle of a carbon chain has O and a double bond is generally referred to as a carbonyl group. At 1670–1780 cm^−1^, the C=O carbonyl group appears, indicating that the peak of the carbonyl group is stronger after surface oxidation. This means that several C=O double bonds are formed through surface oxidation. A strong peak shows that most of the oxygen uptake is bonded to the carboxyl structure, which strongly bonds to the aromatic elements. After the activation, the peak of the carbonyl group becomes smaller. The electronegativity of O is larger than that of C at C=O, so that the π-electron is biased toward O. However, the π-electrons are only slightly biased. Therefore, the carbonyl group has a strong affinity to water molecules and easily reacts, so that the pitch is activated and brought out with O. At 1600 cm^−1^, the double bond region of aromatic C appears. As the surface oxidation temperature increases, the peak becomes sharper, but at 360 °C or higher, the peak becomes smaller. This shows the double bond structure of C resulting from surface oxidation, but the gasification reaction was enhanced at excessively high reaction temperature and the structure collapsed owing to C being eliminated by the formation of CO and CO_2_. Figure 2 and Table 1 prove that surface oxidation has resulted in the combination of low volatile matter. The aliphatic structures retained through the surface oxidation are gasified with steam and are eliminated in the form of CO or CO_2_ to form pores. Surface oxidation is advantageous for the formation of high specific surface area and mesopores^29–32^.

**Figure 5:**
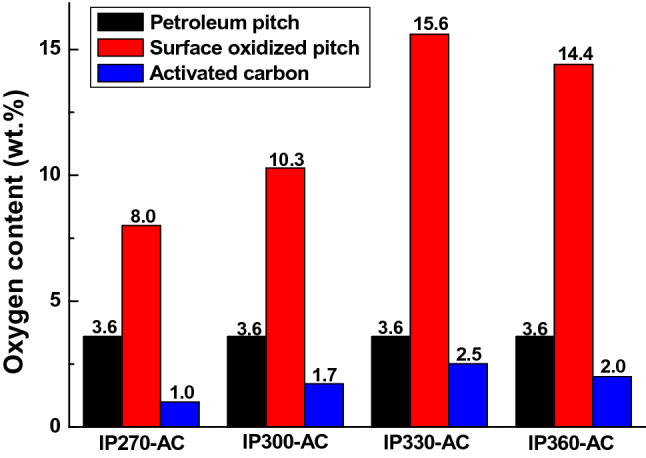
Oxygen contents of surface oxidized pitches and activated carbons from petroleum pitch.

Figure 5 shows the change in oxygen content with surface oxidation and activation. The amount of oxygen in IP270 increased from 3.6 to 8.0 wt.% during surface oxidation and decreased from 8.0 to 1.0 wt.% during activation. The amount of oxygen consumed in the activation process was greater than the amount of oxygen uptake, which indicates that the original pitch consumed oxygen through the activation reaction. Oxygen consumed in this process reacts with the surface of the pitch to form pores and contributes to the increase in the specific surface area. As the amount of oxygen uptake through surface oxidation increases, the amount of oxygen consumed during the activation also increases. In particular, as mentioned in Table 3, mesoporosity increased in the order of IP270 < IP300 < IP360 < IP330, which showed the same trend as the amount of oxygen consumed during activation. Generally, applying a high temperature of more than 800 °C to activate a pitch without surface oxidation causes the volatile matter of pitch to run out and the pitch is harden, making it difficult to form a pore caused by steam and make activated carbon with a large specific surface area. However, the surface oxidized pitch is combined without the release of the low molecular substance, the volatile matter, through the infusilibization by oxygen, which runs out in the form of low molecular structured the volatile matter, CO and CO_2_, making it relatively easy mesoporous activated carbon. The volatile matter of petroleum pitch is 52% used in this experiment, and if the activation is carried out without surface oxidation, the volatile matter is drained out and the pitch is coking. This makes the surface very hard, and activation by steam is very difficult. However, the surface oxidation reaction is bound by the oxygen uptake without releasing low molecular substance, the volatile matter of pitch. When activation energy is applied to this bonded pitch, the bound oxygen is cut off and the surrounding carbon and volatile matter go out together, forming a mesoporous activated carbon with large pores.

**Figure 6:**
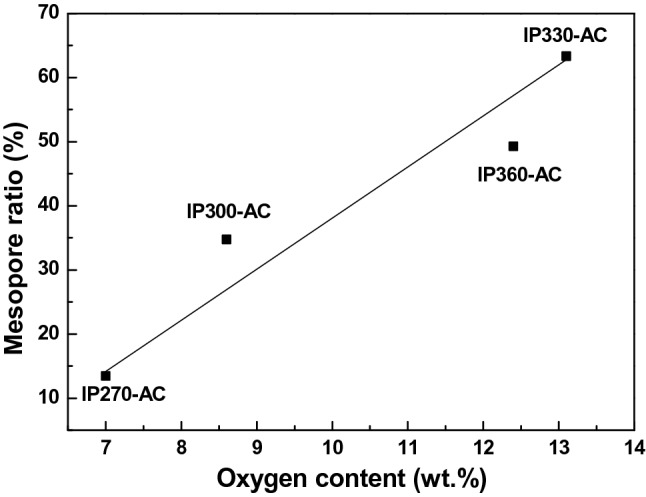
Correlation between oxygen content and mesopore ratio of activated carbons.

Figure 6 shows the effect of oxygen content on the formation of mesopores. Oxygen was consumed in the form of CO or CO_2_ during activation, and pores were formed on the surface of the pitch to result in mesoporous activated carbon. It was confirmed that the amount of oxygen uptake and the ratio of mesopores were proportional. The oxygen content of IP330 was the largest at 13.1 wt.%, and the mesopore ratio was the highest at 63.35%. It was confirmed that the amount of oxygen uptake during surface oxidation is very important to produce activated carbon with high mesopores.

## Conclusions

The thermal stability of the surface oxidized pitch was significantly altered according to the surface oxidation temperature. In addition, as the amount of oxygen consumed through surface oxidation increased, the amount of oxygen released during the activation also increased. Oxygen was introduced in the pitch along with lactone or carbonyl group through surface oxidation, and the oxygen uptake in the activation produced mesoporous activated carbon with a high specific surface area. Low surface oxidation temperature does not ensure an adequate amount of oxygen uptake, whereas high surface oxidation temperature causes an excessive reaction and increases the amount of oxygen emitted. Therefore, it is important to prepare highly mesoporous activated carbon at the appropriate surface oxidation temperature of 330 °C. IP330-AC had a mesopore ratio of 63.35% and a specific surface area of 1811 m^2^ g^−1^. In addition, the mesopore ratio of IP330-AC was approximately 491% higher than that of commercial activated carbon P60 with a similar specific surface area. The oxygen content and mesopore ratio increase proportionally, and a large amount of oxygen is required to produce highly mesoporous activated carbon. It was confirmed that surface oxidation is a very useful method for the preparation of mesoporous activated carbon.
